# Health and care workforce-driven transformations can improve workforce stability and strengthen health system resilience

**DOI:** 10.1093/eurpub/ckag185

**Published:** 2026-10-13

**Authors:** Ellen Kuhlmann, Gabriela Lotta, André Beja, Viola Burau, Olga Gershuni, Zuzana Kotherová, Klaas Stek, Marius-Ionuț Ungureanu, Tiago Correia

**Affiliations:** Institute for Economics, Labour and Culture, Goethe-University Frankfurt, Frankfurt, Germany; WHO Collaborating Center on Health Workforce Policies and Planning, Instituto de Higiene e Medicina Tropical, Universidade Nova de Lisboa, Lisbon, Portugal; Department of Public Administration, Getulio Vargas Foundation, São Paulo, Brazil; WHO Collaborating Center on Health Workforce Policies and Planning, Instituto de Higiene e Medicina Tropical, Universidade Nova de Lisboa, Lisbon, Portugal; Global Health and Tropical Medicine, GHTM, Associate Laboratory in Translation and Innovation Towards Global Health, LA-REAL, Instituto de Higiene e Medicina Tropical, IHMT, Universidade Nova de Lisboa, UNL, Portugal; Department of Public Health, Aarhus University, Aarhus, Denmark; Department of International Health, Care and Public Health Research Institute (CAPHRI), Faculty of Health, Medicine and Life Sciences, Maastricht University, Maastricht, The Netherlands; WHO Collaborating Centre on Public Health Leadership and Workforce Development, Maastricht University, Maastricht, The Netherlands; Institute of Public Health and Medical Law, First Faculty of Medicine, Charles University, Prague, Czech Republic; Institute of Sociological Studies, Faculty of Social Sciences, Charles University, Prague, Czech Republic; European Lab for Innovative Purchasing and Supply, University of Twente, Enschede, The Netherlands; Department of Public Health, Faculty of Political, Administrative and Communication Sciences, Babeș-Bolyai University, Cluj-Napoca, Romania; Center for Health Workforce Research and Policy, Faculty of Political, Administrative and Communication Sciences, Babeș-Bolyai University, Cluj-Napoca, Romania; WHO Collaborating Center on Health Workforce Policies and Planning, Instituto de Higiene e Medicina Tropical, Universidade Nova de Lisboa, Lisbon, Portugal; Global Health and Tropical Medicine, GHTM, Associate Laboratory in Translation and Innovation Towards Global Health, LA-REAL, Instituto de Higiene e Medicina Tropical, IHMT, Universidade Nova de Lisboa, UNL, Portugal

## Introduction

Changing population health and new security threats require a resilient health and care workforce, yet health workers are exhausted, labour market capacities are decreasing, and a coherent workforce strategy is still lacking in most European countries. In this situation, workforce-driven stability and resilience may emerge bottom-up. However, these capacities are not well understood by health policy. Instead, health and care workers are mostly perceived as critical infrastructure and as a cost factor for health systems, obscuring the view of both the needs of individual health workers and their active role in health systems.

This Viewpoint argues that the transformational capacities of the health and care workforce not only need greater attention but also require a new mindset of workforce policy. Bottom-up workforce-driven transformations are crucial for innovating health systems, supporting the implementation of policy reform and advancing equity and inclusiveness in the workforce itself and in healthcare provision. They can strengthen workforce stability, health system resilience and trust in governments and create co-benefits for other sectors. To unlock these capacities, workforce policies must shift from short-term technical crisis solutions towards a coherent strategy that looks beyond headcounts and acknowledges, in a comprehensive and intersectional way, the person behind every health worker, recognizing in most cases a woman and often a migrant.

By focusing on the workforce as an active player and an important pillar of health systems, this Viewpoint highlights new capacities for workforce stability and health systems resilience, calling for policy shifts from crisis towards capacities and implementation [1–3]. The Viewpoint introduces changes driven by knowledge and experience of frontline workers, highlights transformational effects in health systems, and explores supportive governance structures for these capacities to flourish.

## Health and care workforce as an active stakeholder and innovator of health systems

Workforce-driven capacities are highly flexible, and the transformative effects in health systems are diverse and context-dependent. Five major transformational roles can be identified, although these may not be exclusive.

### Policy implementers

Primary care workforce reforms in Denmark and Finland illustrate that health workers’ perceptions matter for implementation. Stakeholder inclusion at an early stage of reform can reduce resistance and support the building of a sustainable primary health care workforce [4]. On the other hand, perceived misalignment with the realities of frontline workers may cause demoralization and potentially worsen shortages if health policy does not respond to the concerns [5].

### System transformers

Community health workers provide an example of comprehensive bottom-up transformations towards stronger community-centred and primary healthcare supporting vulnerable populations; for example, maternity care for migrant women and children, HIV/AIDS prevention, or support for victims of violence including sexual violence [6].

### Workforce stabilizers

Health workers seek to adapt and transform their working routines to respond to growing pressures, improving self-efficacy to protect their mental health and prevent stress and burnout as one of the root causes of the workforce crisis [7]. These emerging *ad hoc* bottom-up capacities for workforce stability were observed, for instance, among general practitioners in Ireland [8] and globally during the coronavirus disease COVID-19 pandemic.

### Organizational bridge-builders

Health frontline workers can act at the meso-level of health systems, connecting different professional groups and provider organizations, including public and private sectors. These capacities are particularly important in team-based provider settings and at the crossroads of healthcare and administrative or technical provider groups, such as procurement [9].

### Social inclusion facilitators

Capacities for social inclusion, equity and gender equality may emerge from new professional groups and organizational settings, such as community care workers [6], nurse-led primary care for refugees in Germany [10], or intercultural care workers in Czechia improving access to healthcare for Ukraine refugees after Russia’s full-scale invasion [11].

Workforce-driven innovations stretch beyond short-term effects of mitigating shortages and facilitating policy implementation and provider management. Health and care workers can mobilize self-efficacy [12] and self-governing capacities to actively design and implement innovations in health systems. Transformative powers are strongest if diverse capacities converge and create a hub of innovation, as illustrated by community health workers strengthening community-centred policy approaches, social inclusion and gender equality [6], or nurse-led care strengthening task shifting, primary health care, new organizational models and access to care for vulnerable groups [10].

## Translating workforce capacities into health system resilience

Health and care workers can ensure workforce stability and support resilient health systems in complex ways, but they cannot do the job of governments. Health policy must create governance architectures that are capable of stimulating and supporting workforce-driven actions and integrating transformational capacities strategically into health policy agendas to achieve the desired outcomes. Governance research and theories [13] offer a broader framework for assessing the connections between bottom-up and top-down capacities and their effects, while meso-level approaches specify transformations in health system contexts.

Research into professions, for instance, shows that governments in the past have used and nurtured the capacities of high-level professions strategically, mostly medicine, but hampered others [14]. Medical professions are integrated in European health systems, serving as mediators between the state and the citizens, policy implementors and service organizers. These capacities are strongest in social health insurance systems that assigned statutory rights to medical professions, but also matter in national health services and mixed systems. However, elitist profession-based policies may reinforce the workforce crisis, for instance, if the medical profession hampers integrated skill-mix policies, teamwork, or new roles for qualified nurses.

Street-level bureaucracy, with its focus on frontline workers and bottom-up innovation, offers a broader approach. It places the linkage of upward (bureaucracy) and downward (street level) dynamics in a wider governance architecture and draws on available practical solutions that support realist and actionable policies, including sensitivity for political instability and vulnerabilities [12, 15]. This research can identify adverse effects, for instance, if workers perform tasks for which they are not sufficiently educated and qualified, as well as conditions for effective bottom-up transformations and innovations, highlighting the importance of strong regulatory frameworks for education and practice.

Migrant and refugee health workers in European health systems provide an example of how bottom-up emerging capacities may be used strategically and integrated in stable governance settings, moving beyond an over-simplicist either–or approach on benefits. Romania, for instance, scaled-up investment in medical education and was able to increase the number of international students, generating revenue, improving the quality and competitiveness of education, expanding labour market capacities and creating a new recruitment pool.

Transformational capacities may also flourish *ad hoc* in uncertain political contexts with vulnerable or collapsed infrastructures. Empirical evidence is mostly taken from countries in the Global South [12, 15], yet increasingly relevant in Europe, as most clearly observed in Ukraine after Russia’s full-scale invasion: strong increases in demand for health workers and new competencies intersect with severe decreases, especially in combat regions and specialized professions [16]. In this situation, Ukraine’s healthcare provision, usually defined by governance frameworks and infrastructures, increasingly shifts towards volatile and hybrid models built on bottom-up workforce driven solutions. For instance, nurses take over coordination and mental health physicians rehabilitation tasks; infectious diseases from the past, such as rabies, recur, and animal health issues spill over into the health system, challenging existing prevention tools; and new digital technologies and platforms are used to support psychosocial care and mental health resilience.

All these tasks require high flexibility and new knowledge for which the health and care workers are not sufficiently educated and trained. Ukraine illustrates how workforce-driven capacities ensure healthcare provision under conditions of vulnerable governance and infrastructures. Yet, it also highlights lessons to be learned for improving preparedness and protection of the workforce, and strengthening public health [16, 17] and health security [18]. Governments in Europe and beyond should urgently learn these lessons.

## Conclusions and policy directions

The health and care workforce policy-crisis calls for radical transformations in health policy and systems, moving the debate towards capacities and workforce-driven innovation. Four major strategic policy shifts are indicative to fully enhance and integrate the transformative workforce capacities.

- Establishing a multi-level participatory governance architecture that connects bottom-up capacities and self-efficacy of frontline workers with top-down strategic guidance, using existing knowledge and actionable policy solutions more coherently and effectively.
- Shifting health systems and the organization of care towards community- and primary care-centred approaches that are better able to stimulate bottom-up innovation and integrate workforce-driven knowledge strategically into wider health policy.
- Shifting workforce policy from technical approaches on human resources as part of infrastructures towards health and care workers as persons and the workforce as an essential pillar of health systems security and resilience.
- Improving data and research on workforce-driven innovation to create governance architectures capable of integrating transformative capacities, while ensuring regulation and strategic oversight of workforce stability and health system resilience.

## Data Availability

No original data were collected for this article.
